# Using Behavioural Reasoning Theory to Explore Reasons for Dietary Restriction: A Qualitative Study of Orthorexic Behavioural Tendencies in the UK

**DOI:** 10.3389/fpsyg.2021.685545

**Published:** 2021-07-08

**Authors:** Elina Mitrofanova, Elizabeth K. L. Pummell, Hilda M. Mulrooney, Andrea Petróczi

**Affiliations:** Faculty of Science Engineering and Computing, Kingston University, Kingston upon Thames, United Kingdom

**Keywords:** orthorexia nervosa, restricted diet, healthy eating, eating disorder, food purity, food choices, food ethics

## Abstract

Orthorexia Nervosa (ON) has gained increased attention in academia since 1997. However, like other “Exia” conditions, there is debate around its inclusion in the Diagnostic and Statistical Manual of Mental Disorders. This study aimed to examine the experiences of those following a diet indicative of ON in the United Kingdom. This information is essential to the development of diagnostic criteria and classification of ON. Behavioural Reasoning Theory was used to explore reasons contributing to the development of ON. Ten individuals (two males and eight females), aged 23–35 years, took part in semi-structured interviews. Inductive thematic analysis was used to analyse the results. Four themes emerged from the data—journey, social, rules/control, and ethical considerations which highlighted contributing factors to the development of ON behaviours, the impact of these behaviours on individuals' social lives, and a strive for control. These findings are the first to suggest that ON involves a more complex set of behaviours than current definitions and proposed diagnostic criteria suggest and highlight the need to consider a variety of reasons for restricted diet when developing diagnostic criteria, screening tools, and classification in the DSM.

## Introduction

Orthorexia Nervosa (ON) refers to an extreme preoccupation with healthy eating (Bratman, 1997). Diagnostic criteria for this condition have been proposed by several authors (Cena et al., 2019), with agreement yet to be reached. This is not surprising since much of the existing research has focused on establishing prevalence (Alvarenga et al., 2012; Asil and Sürücüoǧlu, 2015; Almeida et al., 2018), investigating the association between ON and other psychiatric conditions (McComb and Mills, 2019), and developing and validating diagnostic tools despite uncertainty around the criteria (Gleaves et al., 2013; Barrada and Roncero, 2018; Chard et al., 2019). The Diagnostic and Statistical Manual of Mental Disorders, fifth edition (DSM-5; American Psychiatric Association, 2013) includes a diagnostic category “Other Specified Feeding or Eating Disorder” (OSFED), which captures atypical presentations of diagnosable eating disorders. ON, like other “exia” conditions (e.g., drunkorexia, bigorexia, pregorexia) is not included under the OSFED classification but recent research suggests the majority of health professionals would welcome its inclusion, with this bringing advantages in treatment options and promoting further research (Ryman et al., 2019). However, it is imperative that any proposed diagnosis be validated via a series of steps (i.e., clinical description, laboratory studies, establishing distinctiveness from other disorders, follow-up and family studies; Robins and Guze, 1970). Despite the growing number of publications, research in the field of ON has not yet managed to reach consensus about the clinical description. Furthermore, understanding of how and why this condition develops is lacking.

With this goal in mind, the authors of the current study sought to investigate the motivation, reasons, attitudes, and thoughts underlying ON. These factors, which result in a change in eating patterns are important to understand as this can predict whether restricting food intake benefits health or leads to pathology (Gulliksen et al., 2017). The role of cognition in changing eating behaviours has previously been explored but not in ON, e.g., intention to eat healthily translated into increased fruit and vegetable intake in a study that applied the Theory of Planned Behaviour to healthy eating (Conner et al., 2002). Further theoretical advancement emphasised the role of reasons for adopting behaviours in Behavioural Reasoning Theory (BRT), suggesting that reasons represent an important dimension linking beliefs, intentions, behaviour, and global motives (attitudes, subjective norms and perceived control). As well as predicting global motives, reasons are expected to influence intention directly. They help individuals navigate the world by providing them and those around them with causal justification for their behaviour (Westaby, 2005). It is hypothesised that to execute a behaviour with confidence people rely on past experiences with the most justifiable reasons. In particular, the theory distinguishes between “reasons for” and “reasons against.” BRT has gained prominence and has been applied to health behaviours (Sahu et al., 2020), including exploring reasons for binge drinking (Norman et al., 2012), using controlled performance and appearance-enhancing substances (Lazuras et al., 2017), and organic food consumption (Ryan and Casidy, 2018).

In this study, BRT was used to explore the reasons, motives, and beliefs behind orthorexic tendencies. ON was hypothesised to involve restricting one's dietary intake based on the quality of food (Dunn and Bratman, 2016). However, results of existing studies suggest that cognitions leading to food restriction based on “purity” may be more complex than proposed diagnostic criteria (Cinquegrani and Brown, 2018; Greville-Harris et al., 2020; Fixsen et al., 2020; McGovern et al., 2020; Valente et al., 2020). For example, Barthels et al. (2020) discovered that ON tendencies among vegans were associated with health, aesthetics and healing motives, while ethical reasons (e.g., concern for animal welfare) served as protective factors against developing ON eating behaviours. Considering BRT's focus on reasons as predictors of behaviour, exploring orthorexic tendencies and associated cognitions could provide a more accurate picture of the development and maintenance, and therefore classification, of ON. Most research to date using BRT has employed quantitative methodology (Sahu et al., 2020), and the absence of any pre-existing theoretical framework in the exploration of ON may lead to the replication of findings without the addition of novel insights (Andersen and Kragh, 2010). In this study, drawing upon BRT offers a way to explore behaviour and reasons for ON in an in-depth manner.

The need for qualitative approaches to ON has been identified by previous research (Håman et al., 2015). To date, only six studies have attempted to explore experiences of people with possible ON using qualitative methodology (Cinquegrani and Brown, 2018; Greville-Harris et al., 2020; DeBois and Chatfield, 2020; Fixsen et al., 2020; McGovern et al., 2020; Valente et al., 2020). Food intolerances and preoccupation with beauty ideals were shown to predict a lower preoccupation with healthy eating, while worry about future chronic disease was predictive of higher preoccupation with healthy eating (Valente et al., 2020). In contrast, a study by Fixsen et al. (2020) reported the quest for an ideal body is one of the motives for following a restricted diet among individuals identifying that healthy eating had taken over their lives. McGovern et al. (2020) interviewed individuals self-identified as recovered from ON and reported external influences (e.g., familial influences, alternative, and allopathic health professionals) to be key to the onset of ON. However, participants also reported prior or co-occurring EDs (i.e., bulimia nervosa, anorexia nervosa, and binge eating disorder), which might have influenced individual's narratives. Common to all existing qualitative studies is external social influence on the development of ON, emphasizing the need for further exploration to determine other contributing factors. Considering the negative experiences reported, whether related to a cycle of fear and avoidance of foods experienced by bloggers (Greville-Harris et al., 2020) or anxiety, depression and even suicidal ideation reported by recovered orthorexics (McGovern et al., 2020), the need for development of effective treatments to address the negative consequences of ON is growing (Walker-Swanton et al., 2020). Before any progress can be made however, clearer understanding of ON as a health compromising behaviour is needed. No existing qualitative studies, to date, have explored ON through the lens of health behaviour theory. This article therefore aims to contribute to the current qualitative understanding of the reasons/risk factors, beliefs, and motives of individuals with possible ON for following their diet from the perspective of BRT.

## Materials and Methods

### Participants

Ten individuals (eight females and two males) aged over 18 years were interviewed (mean age of participants 28.3 years). Six individuals were employed in the fashion industry, one studied nutrition and sport science, one was a psychology researcher, one was self-employed, and one was a paralegal. The last two participants were recommended by other participants based on their similar eating restrictions, which reflected possible ON symptoms defined by Dunn and Bratman (2016). The lack of officially recognised diagnostic criteria and tools makes recruitment of participants to studies attempting to explore ON challenging. In this study, participants were selected based on observations of eating behaviours and attitudes made by the research team. All participants claimed to adhere to a “healthy” diet, expressed very specific beliefs about foods' properties (e.g., “onion is bad for my chakras”), reported little variation in their day-to-day dietary consumption, and expressed a sense of satisfaction reliant on adherence to the chosen diet. Table 1 reports participants' demographic characteristics, employment status, and self-identified dietary preferences.

**Table 1:** Demographic measures and self-reported dietary preferences of participants (*n* = 10).

Participant ID^*^	**Sex**	**Age (years)**	**Ethnicity**	**Self-reported dietary preferences**	**Employment**
Ebou	Male	32	Black African	Halal	Self-employed
Matt	Male	24	White British	None	Student/Carer
Anna	Female	25	Other White	Vegan	Psychology researcher
Em	Female	35	Other Asian	Vegan	Paralegal
Sarah	Female	29	Other Asian	Vegetarian	Model
Lynn	Female	24	Other White	Vegan with occasional addition of chicken.	Model
Silvia	Female	23	White British	Vegan with occasional consumption of eggs	Model
Elizabeth	Female	33	White British	None	Make-up artist
Rafaela	Female	27	Mixed	None	Retail (high end fashion brand)
Cat	Female	31	Other White	None	Model

* *Pseudonyms are used throughout*.

### Recruitment

The recruitment strategy involved a purposeful snowball sampling technique. Ten individuals were approached and asked to participate in this study. When recruited, participants were given an information sheet using neutral language with no mention of ON due to the lack of formal diagnostic criteria for ON. No exclusion criteria were applied by ethnicity, occupation, or sociodemographic status. Exclusion criteria included current or previous eating disorders or other psychiatric diagnosis, inability to speak English, and age under 18 years. The study was conducted in the UK.

### Procedure

This study was approved by the SEC Faculty Research Ethics Committee at Kingston University. Eight interviews were conducted face-to-face in a location of the participant's choosing. Two interviews were conducted via Skype since participants were abroad at the time but followed the same format as in-person interviews. A disclaimer was read aloud to all participants that assured them of confidentiality, encouraged them to speak without fear of judgement, and outlined the intention to have a discussion about their personal experiences, reasons, motivation and feelings that they assigned to their relationship with food. We emphasised participants' right to withdraw from the interview at any time. Interviews were open-ended and audio-recorded; the average length was ~1 h (total of 8.08 h recorded). Interview guides were based on published ON literature (Brytek-Matera, 2012; Varga et al., 2013; Håman et al., 2015) and explored the influences of dietary practices on social and professional domains, and perceptions and meanings assigned to the concept of a “healthy” diet. Part one of interview guide included questions about the meanings and perceptions of healthy diet and healthy lifestyle, part two asked about the development of their current dietary practices, part three asked about the beliefs surrounding inclusion and exclusion of foods from the diet and practices of food selection and preparation, part four explored participants' perceptions about the impact of their choices on the social lives, part five asked about participants physical activity. In line with the BRT, interviews explored reasons for developing and adhering to current eating practices. In particular, because of the BRT's focus on “reasons for” and “reasons against,” participants were asked to explore the “reasons for” initiation and maintenance of their chosen dietary practices. Participants were also asked to explain their “reasons against” including certain foods in their diets and the impact of their dietary restrictions on other domains of their lives. Interviews were conducted and transcribed verbatim by the first author (EM) using pseudonyms selected by the participants. Recruitment and data collection took place from June to September of 2018.

**Figure 1:**
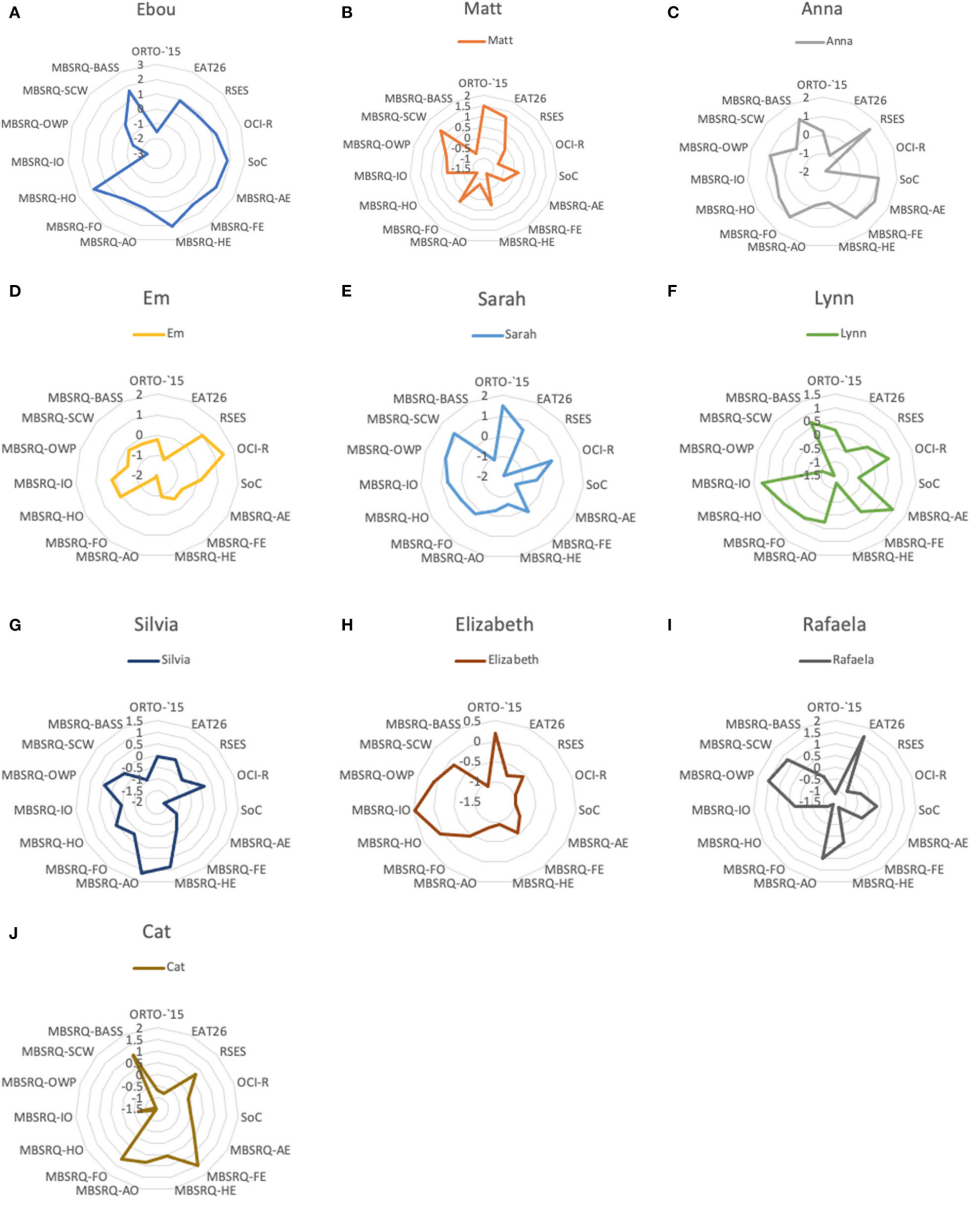
**(A)** Ebou's scores on psychometric measures. **(B)** Matt's scores on psychometric measures. **(C)** Anna's scores on psychometric measures. **(D)** Em's scores on psychometric measures. **(E)** Sarah's scores on psychometric measures. **(F)** Lynn's scores on psychometric measures. **(G)** Silvia's scores on psychometric measures. **(H)** Elizabeth's scores on psychometric measures. **(I)** Rafaela's scores on psychometric measures. **(J)** Cat's scores on psychometric measures. The chart reflects standardised values for all measures. ORTO-15 (Donini et al., 2005). EAT-26, Eating Attitudes Test (Garner et al., 1982). RSES, Rosenberg Self-Esteem Scale (Rosenberg, 1965). OCI-R, Obsessive-Compulsive Inventory-Revised (Foa et al., 2002). SoC, Paulhus's Spheres of Control Scale (Paulhus, 1983). MBSRQ, Multidimensional Body-Self Relations Questionnaire (Cash, 2015); MBSRQ-AE, Multidimensional Body-Self Relations Questionnaire Appearance Evaluation subscale; MBSRQ-FE, Multidimensional Body-Self Relations Questionnaire Fitness Evaluation subscale; MBSRQ-HE, Multidimensional Body-Self Relations Questionnaire Health Evaluation subscale; MBSRQ-AO, Multidimensional Body-Self Relations Questionnaire Appearance Orientation subscale; MBSRQ-FO, Multidimensional Body-Self Relations Questionnaire Fitness Orientation subscale; MBSRQ-HO, Multidimensional Body-Self Relations Questionnaire Health Orientation subscale; MBSRQ-IO, Multidimensional Body-Self Relations Questionnaire Illness Orientation subscale; MBSRQ-OWP, Multidimensional Body-Self Relations Questionnaire Overweight Preoccupation subscale; MBSRQ-SCW, Multidimensional Body-Self Relations Questionnaire Self-Classified Weight; MBSRQ-BASS, Multidimensional Body-Self Relations Questionnaire Body Areas Satisfaction.

**Figure 2:**
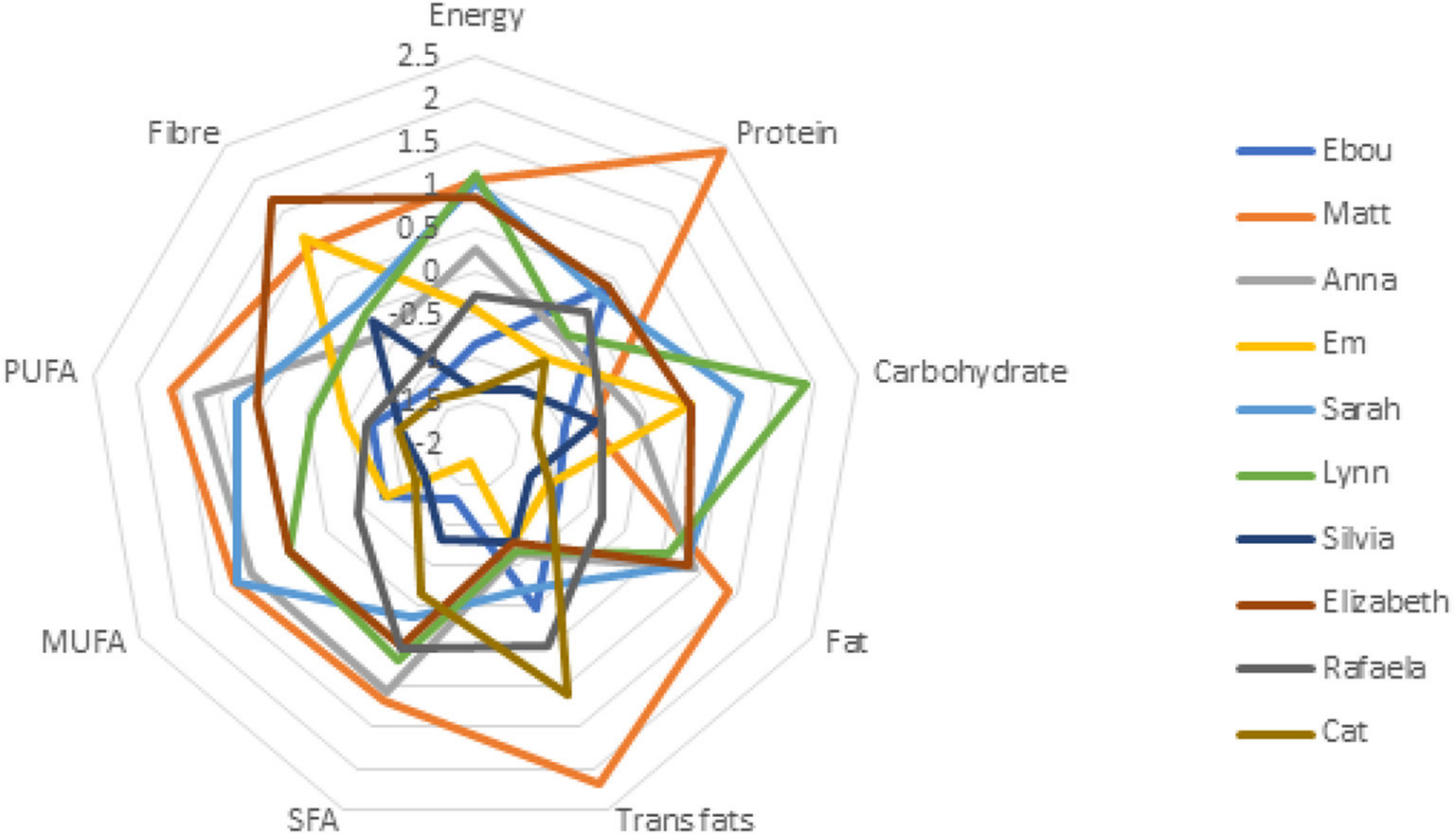
Nutritional intakes were standardised. Full nutritional intakes are available in Mitrofanova et al. (2020). SFA, Saturated Fatty Acids; MUFA, Mono-unsaturated fatty acids; PUFA, Poly-unsaturated fatty; acids; PUFA, Poly-unsaturated fatty acids.

Participants also completed a battery of psychometric measures and provided 24-h recalls of dietary intake. Figure 1 shows patterns of participants' scores for psychometric measures, and Figure 2 demonstrates the disparity in intakes of specific nutrients to provide context for the qualitative data. 24-h recall assessed nutritional intakes of one weekday for each participant. No specific measures of reliability or validity were undertaken in this study, but other studies have shown lower levels of variability and EI under-reporting using 24 h recalls compared with other methods of dietary intake assessment, albeit with differences by gender (Burrows et al., 2019). Detailed data from psychometric and nutritional assessments are published separately (Mitrofanova et al., 2020). This article reports only the analysis of the driving forces behind their qualitative attribute-based food restriction practices.

### Data Analysis

Thematic analysis as defined by Braun and Clarke (2019) was used to analyse the interview data. This is a flexible method that may be used by researchers relying on differing philosophical underpinnings. The study was designed following a paradigm of pragmatism. This position suggests that ontological essence lies in actions and change; humans are actors in the constantly changing world (Goldkuhl, 2012). Pragmatism does not dictate or limit the use of different methods in research, but adopts a pluralistic position (Goles and Hirschheim, 2000). In this article, thematic analysis was used not only to describe the actions and cognitions of individuals, but also to understand the changing nature of reasons in a world that is in a state of constant becoming.

Thematic analysis was used inductively without assigning preconceived notions of ON. The first author (EM) repeatedly read and became familiar with the full data set. Atlas.ti (ATLAS.ti Scientific Software Development GmbH) software was used for open thematic coding to identify “meaning units” according to the “bottom-up principle.” Codes related by their semantic and conceptual readings were then grouped into sub-themes. Sub-themes were continually collapsed together to generate larger themes. Final codes and sub-themes were reviewed and agreed upon with the second author (EP).

The consolidated criteria for reporting qualitative research (COREQ) tool was used to monitor and report important aspects of the research process (Tong et al., 2007; see Supplementary Table).

## Results

Four themes were identified from the data that reflect the reasons for following the self-defined “healthy” diet: “journey,” “rules/control,” “social,” and “ethical considerations.” Table 2 presents themes, sub-themes and examples of participants' quotes.

**Table 2:** Themes, sub-themes and example quotes of participants.

**Theme**	**Sub-themes**	**Example quotes**
Journey	Re-evaluation of what is healthy	“They're such set ways like I think we been brought up with being told that you get strong and amazing and it's the only way, to drink milk for example, like my dad would have a large pint of milk and I think it looks bizarre, like bec. but that's the way we've been brought up and I think a lot of it comes down to, like the welfare years in Sweden when they wanted to boost the whole milk industry um… so obviously they gonna tell people, politicians are gonna tell people that milk is great.” (Lynn)
		“A lot of my diet was around just protein back then. So, as I got older, I realised, I've done more reading, watched more videos, followed more… like bodybuilding and weight training type of um… like… art schools and stuff, and… I kind of just made little tweeks through the years and getting better and better at it.” (Matt)
		“I mean I just like you know with time experience I just realised that um… you know like… it's more and more information about dieting and stuff and it's so… if before for example it was so easy to say you know for example what's your mother said or your dad said: ‘eat that, or don't eat that' where is now so many information so I guess it's only with experience I started to feel traits, what exactly works for me and for my body.” (Cat)
	Fluctuating eating patterns	“yeah, so maybe I was pescatarian between 30 and 33 and then vegetarian and 4…5 months and then… vegan, more or less vegan, I mean I did realise some product did have egg in it or milk but mostly, mostly vegan” (Em)
		“My interest has grown so much in five years, like more and more, more food and I think I've experimented a lot, I've done like raw vegan, I've done vegetarian, I've done um… now I'm like mainly vegan, at the moment I'm adding a bit of chicken and fish to get enough protein um… Yeah.” (Lynn)
	Life changes	“I moved to London five years ago and I used to be… really healthy but without even know it… that I was healthy and I moved to London and I saw it as a… almost like a ‘Candyland', everything was a temptation and there is so much sugar in everything here and you just… you are twenty and you think that you can just eating without suffering any consequences so… and sugar is addictive so you start eating sugar and then eating the sugar is now a problem and you feel weak and you know, your skin is not good, but you know, you still don't associate it with the sugar, you justify yourself because you want more of the shit, want to indulge more. So it took me… quite a long time… to identify the problem and… fix it.” (Rafaela)
		“I thought I want to explore, like, all the countries with everything they give and um… the food is also, like, very important part of the culture, so I wanted to try everything” (Anna)
		“I moved to America, pretty much like, what… year… year and a half ago. And pretty much, 95% they put in corn syrup. In everything like, in everything like that you know, you see pastries, or like, you know like different types of bread or like, popcorns, or whatever snacks. You know, you really have to look for it, and some of them, like un… un… the things that you wouldn't expect sugar to be there, like rice… like rice cakes something which is normally should be just rice” (Cat)
	External influences	“It was a cousin of mine, it was a cousin of mine. And he introduced me to… exactly I was outside at his house and he was having a lot of fruit and veg there and he brought some over and… It tasted so nice! Like the fruit, it just tasted so… the apples and everything tasted so juicy and nice and it weren't like big or anything like that it was just a normal sized apple and all these other fruits, and… I said to him where did you get these from? and he told me, listen, it's not a rubbish that you are used to. This is organic.” (Ebou)
		“I've always been pretty healthy. Um… I read a book by a nutritionist called Kimberly Snyder maybe about three years ago. And that's when I got more into like healthy vegan eating.” (Silvia)
		“My friend did power lifting, and he was 16 and did power lifting, he was really good at it. A big chap as well for 16 years old, and he actually… he broke a record and he managed to lift some like 200 kilos. Deadlift, at 16! And um… I kept seeing him each week, each weekend he would come up and he would always bring all this food with him, he had like… three different meals just for… just for 4 h of… so he got three meals for the whole evening he's at my house with my brother and um…yeah I started… he helped me get into it and trained with me three times a week, Mondays, Wednesdays, and Fridays.” (Matt)
Social	Influence of participants' diets on others	“I can't eat where he eats, my partner! So, yeah, and now he thinks that I'm a food snob so, yeah.” (Elizabeth)
		“Most people are actually not even just accepting but… like, um… they actually think it's exciting to eat something else at Christmas, you know what I mean, it's kind of boring to do the same thing every year isn't it?” (Lynn)
		“My dad is really nice, he… because my mom doesn't cook at all ever. Like she can't, she can't cook, like really she can't. Um… my dad, my dad he is quiet… he is really like kinda into it. If he knows I am coming he'll make something vegan, something vegetables.” (Silvia)
		“So… um… then you feel like you are difficult, you know, and you are difficult for waiters… waitresses… for your friend you're difficult, for people at work so… I used to pack my lunch, you know… for… breakfast and lunch” (Cat)
		“But he is a… he likes meat and… so… I think because of me I think he eats less meat. Because you know… otherwise he has to cook two meals.” (Sarah)
	Misunderstood by social circle	“Maybe they think, poor girl, she can't even eat meat. Oh yeah! Yeah! Don't you miss the taste of meat? People definitely talk, gossip, at work, yeah… that's a bit annoying and people do think… my mom probably thought some form of eating disorder. cause they don't understand.” (Em)
		“They would question it because I think my mum would be worried that I'm not getting enough…, enough food and enough nutrients that I need.” (Lynn)
		“I can see the family relationship can be tricky like siblings, for example, oh yean she has to have one dish just to be made for her, and that can be difficult.” (Sarah)
	Organising role of dietary choices	“I'll just want maybe at least once a week or once in two weeks I go vegan buffet with other vegan friends.” (Em)
		“Occasionally, only when my other sister, I have a sister who has 3 kids, and they come, we… we sit together but I'll still eat my own food.” (Em)
		“Like with my friends at home, I'm living in a shared living with two other girls and they are at least vegetarian too. And one is also um… she wants to be vegan and like, only raw vegan, so that's a little bit more special than me. Um… so at my home it is perfectly fine when we want to eat together. And when we go to restaurants it's quite easy as well, because there is always at least a salad or something. And… and… yeah, most of my friends they know how I eat.” (Anna)
		“Since I moved to London five years ago, Christmas is not something that I do with my family anymore, it's something that I do with my close friends in London and you know because… we are all in the same box.” (Rafaela)
		“That's not a problem, because like, I know what I want for me, but I would never tell any other people how to behave or what to do, like people have to make their own experience and um… yeah. And if they… they have to live they want to and I am like this I make my own experiences and I feel better this way.” (Anna)
Rules/Control	Dietary restrictions	“it's um…chemicals that's um… like hormones inducing chemicals to make plants grow it's extra, it's unnecessary, the whole point with the organic to… to what I believe is to keep it as clean and as natural as possible I mean, in an ideal world I'd have my own vegetable garden, so it's kind of, so that's what I'm always trying to strive for.” (Elizabeth)
		“Junk food, like the McDonalds, and the KFCs, and like processed meat; it's packaged, packaged or tinned meat or fruit and veg. That's for me what I consider processed and you can even added on to sort of extend the shelf life so when there are chemicals in it to extent shelf-life. All those things I try to stay away from as much as I can.” (Ebou)
		“Things that could be meat, or could be veggies from like local um… small stores and it delivers to you and I know that is 100% organic and it's like a… it's a peace of mind. My mind is at peace so, and I know I don't have to worry and I know that the meat I'm having delivered, it wasn't, you know, like pushed with steroids or something.” (Rafaela)
		“Like anything says “diet” cause I heard when it says “diet” they have um… they put something instead of sugar which is human body can't digest it so it stays in the body so it's better to have something which is… rather than processed, also I find like a lots of apples, for example, all waxed outside, so it's already um… not organic so you basically eating that [wax] doesn't help digestion I think.” (Sarah)
		“I can't remember, they put something, which reduces the coffee and the fat, chemicals and stuff like that. But then, there is no research's been done into the long term effects of these chemicals in the food after, that are left behind, so I just sort of stay clear, stay away from that as well.” (Matt)
	Rigidity	“I don't take risks, so like… I won't just choose somewhere to go, oh, I've heard that's nice, I won't go, oh, I'll go there, cause what happens if I get there and it's not, I won't get what I paid for. It's like, I don't know, I don't try new things either so I'll stick to the same thing all the time that way I know what I'm getting, I know it's gonna be worth it and I will be happy in the end, cause of… I feel if I take risk every now and again, I might end up not enjoying my food and then having to go home and eat more.” (Matt)
		“You know, to be honest, especially with travelling, it's very hard for me. If I travel, for example, to the places that I know, I'm fine. But… if I travel to a new place, I don't know… I really struggle to eat. I have to watch myself because I have this tendency that I stop eating. I wouldn't eat. I know that it's very bizarre cause I would go to the shop and if I don't know or don't recognise the products which are on the shelves… for me it's very hard to try something… yeah. Something new… to eat. So that's why I always end up eating vegetables that I know, because I can always find an apple or um… an avocado… or like you know salad leaves.” (Cat)
		“I try to eat only natural, like, like I don't eat something somewhere else, that's why I cook for myself, so, all the others go here and have lunch and I always bring my own food. So I… so I know what's in there. (Anna)
	Influence of diet on other areas of life	“Because I can't control my body and you need to go toilet and… yeah I thought I'd rather have a strict diet if I can control my appearance and if I can control my body so you know if there is a business meeting and I can't focus and I can't do my job.” (Em)
		“If I wanna have some McDonalds or some bad food, then I'll… I'll just have it. I won't you know, I'll just have it or I'll, I'll put that into my calories kinda thing, so…I have a calorie counter and everything I've told you now meets my calories, so if I want to have a Mc Donalds that day I'll just take one of those meals out put that McDonalds in there.” (Matt)
		“But to be honest, now I have learned to tailor my. and I'm not saying that I choose my holiday based on the food they make, I just tailor my holiday around something that works for me. For example, the next holiday is in the Philippines, but I'm never going to go to Manila because… it's disgusting, and I don't think the food there is any good.” (Rafaela)
	Physical activity	“I'm practicing yoga every day in the morning and in the evening um… and yeah also to be aware of what is around you, like the people and the animals, you know, so it's like yeah… to… to… to really be aware of everything around you and um… and so, it's the food you know? To practice yoga you need good food.” (Anna)
		“I prefer to do something like a class, something more fun, cause sometimes I like run on a treadmill but it's so boring. Like counting down the minutes so you can stop. Whereas dancing is like, enjoyable, I think it's good for your like, you can like, feel happy. Good for your like… psyche.” (Silvia)
		“Um. I mean… um… not really far from park so I can run… um… which is quiet important” (Sarah)
		“Well, ballet normally… um… one and a half hour, and taekwondo is about 2 h. And a class is of course not just hard or anything the class is build up and build up.” (Cat)
Ethical considerations	Ethical concerns for food production	“First of all, can I say that… nowadays I absolutely love markets more than I love supermarkets in the sense that… for me… is not about deprivation it's about… eating everything as long as it's wholesome and it's good food so the market in a way gives me the… the insurance that this food comes from the earth and it's been moved to the closest market and it's been sold as close as possible to the earth. So… what's matter to me is… it's that whatever I put in my mouth, comes from a good place. And then I think… I don't deprive myself: you can eat anything as long as… it's ethically sourced and wholesome, and it's good for you and not full of additives and… you know they don't put a syringe of whatever in my chicken and stuff like that.” (Rafaela)
		“Eating something healthy to me is don't just start from the shelf start from the root. How is it grown, how is it been treated and transported over to the shelves. That's more of a healthy thing for me, personally.” (Ebou)
	Food for survival not enjoyment	“You can survive most of the time being plant-based, so I just prefer to, it's not necessary to kill them! You know what I mean? It's not necessary to… I mean medicinal honey it's good, but… you don't have to take it unless you are so old or something.” (Em)

### Journey

Our participants spoke about their journeys to development of their current habits and beliefs about their chosen diets. Change for all individuals was gradual and was often described as a “transition.” For example Ebou explained:

I didn't automatically just change, so I change my views on it and I started ordering fruit and veg from there [organic delivery company] because it tastes nicer and um… yeah, just since then started looking into it a bit more…

Several factors were indicated as key to initiating the transition. The first was described as re-evaluation of what was thought to be a healthy diet and acceptable food practices by participants' families. This re-evaluation represented a change in cognition from “eat what's on your plate” to exercising agency over food choices. Individuals in this study, although UK residents at the time of data collection, came from various cultural backgrounds. Lynn criticised her parent's beliefs for being based on outdated government guidelines:

They're such set ways like I think we been brought up with being told that you get strong and amazing and it's the only way, to drink milk for example, like my dad would have a large pint of milk and I think it looks bizarre, like bec. but that's the way we've been brought up and I think a lot of it comes down to, like the welfare years in Sweden when they wanted to boost the whole milk industry um… so obviously they gonna tell the people, politicians are gonna tell people that milk is great.

Despite a very different cultural background, Em expressed a similar view about differing perceptions between generations: “I used to think vegans are maybe confused, maybe… because I just literally thought animals can be eaten and they are for us to use… that's my, you know, maybe that's the way of thinking inherited from previous generations.” The gradual change in eating habits was informed by fluctuating eating patterns. Individuals indicated that they had tried several diets before settling on the current one. Lynn explained: “My interest has grown so much in 5 years, like more and more, more food and I think I've experimented a lot, I've done like raw vegan, I've done vegetarian, I've done um… now I'm like mainly vegan, at the moment I'm adding a bit of chicken and fish to get enough protein um…Yeah.”

The second significant influence on individuals' journey was relocating to a different country. Participants experienced a disinhibition in their eating practices. Some, like Anna, abolished their restrictions consciously to experience the new culture: “I thought I want to explore, like all the countries with everything they give and um… the food is also, like very important part of the culture….” However, others found themselves lost without familiar foods and defined their current diet as an attempt to take back control. For example, Rafaela explained:

I moved to London and I saw it as a… almost like a “Candyland,” everything was a temptation and there is so much sugar in everything here and you just… you are twenty and you think that you can just eat without suffering any consequences so… and sugar is addictive so you start eating sugar and then eating the sugar is now a problem and you feel weak and you know your skin is not good, but you know, you still don't associate it with the sugar, you justify yourself because you want more of the shit, want to indulge more. So it took me… quite a long time… to identify the problem and… fix it.

Distrust toward ingredients in foods of the host country was implicated in increased scrutiny of food labels. Sugar and corn syrup were among the additives that raised suspicion, resulting in increased restrictions of foods suspected to contain them. Cat explained:

And pretty much, 95% they put in corn syrup. In everything like, in everything like that you know, you see pastries, or like, you know like different types of bread or like, popcorns, or whatever snacks. You know, you really have to look for it, and some of them, like um… um… the things that you wouldn't expect sugar to be there, like rice… like rice cakes something which is normally should be just rice.

Finally, for most participants the onset of the change in cognitions about “healthy” could be traced to the influence of family, friends or social media. For example, for Em it was a vegetarian friend who refused to socialise with meat eaters: “It was like a social thing, I knew she doesn't like to see meat in front of her, so when I did go out with her, I didn't eat meat.” Em also indicated a social media influencer as her inspiration for switching to a raw food diet at a later stage. For other participants it was a family member: “… he told me, listen, it's not a rubbish that you are used to. This is organic. Since then I started having nicer fruit and veg and I used to only have like brown rice organic… that was it” (Ebou). For Anna it was a yoga teacher that defined the appropriate diet. Interestingly, the rules adopted from the social circle resulted in a change in behaviour toward the social circle itself.

### Social

This theme contains individuals' elaborations about the impact of their chosen diet on their day-to-day interactions and social lives. Most participants acknowledged that prioritising their diet impacts their immediate social circle. Family members cook separate meals at family gatherings (e.g., Christmas). When eating out, participants' views about the impact of their chosen diet differed. On one hand, individuals did not perceive any discomfort their diet might cause to their acquaintances. They identified their diet as an inseparable part of their identity and suggested that their friends were well-informed of their dietary patterns; they would not maintain the friendships if their social circle was not considerate of their requirements. On the other hand, some of the participants reflected that their eating habits had a negative impact on their social relationships. For example Cat suggested:

I mean psychologically it's annoying. Even like to be on a diet what I found is that psychologically, socially it was difficult. You know? Because like, you make a fuss all the time about what you can not eat. You're making an order in a shop, in a restaurant, in a bar, at a friend's place “Oh, I can't eat this. I can't eat that” and you know, when you… when people… of course first time people are fine with this but the second time people start making you comfortable and trying to offer you options, and because you can't eat anything you say no, no, no, no and this is how people start getting annoyed. You know? So… um… then you feel like you are difficult, you know?

Em felt like she was misunderstood by her immediate social circle: “people definitely talk, gossip at work, my mom probably thought some form of eating disorder, cause they don't understand.” This theme is connected to the elaborations offered in the previous one (Journey). Social influences were prominent in descriptions of the onset of the dietary patterns and played a role in social relationships participants chose to form. For example, Anna mentioned that maintaining her diet is facilitated by the people she lives with: “I'm living in a shared living with two other girls and they are at least vegetarian too. So, at my home it's perfectly fine when we want to eat together. And when we go to restaurants it's quite easy as well, because there is always at least a salad or something.” There was a notable disparity between the reported attitudes toward other people's dietary habits and the reported behaviour. For example, the previous quote points toward a behavioural choice of this participant to share accomodation with people following a similar diet but the general attitude reflected diet to serve a function of othering: “I know what I want for me, but I would never tell any other people how to behave or what to do, like people have to make their own experience and um… yeah. And if they… they have to live the way they want to and I am like this I make my own experiences and I feel better this way” (Anna). This reflects an attempt to situate oneself as individual that claims respect for others' choices yet prefers to associate with people following a similar diet.

### Rules/Control

Individuals reported following very specific dietary rules. The four vegan participants differed in their descriptions of veganism. For example, Lynn, despite self-identification as vegan ate chicken and fish, while Anna (also a self-identified vegan) excluded garlic and onion from her diet. Explanations for these very particular rules also differed. Lynn explained that she included animal products because following a strict vegan diet has led to adverse health consequences (hair loss), while Anna explained that onions and garlic were excluded due to these foods' perceived antiseptic properties.

Um… because there are some substances in… in these um. that are… yeah… a little bit like antibiotic so they are… they have influence like in the body, you know people say that it's a good influence but I want to keep my um… my body as natural as possible. That I don't kill anything in my body because if I… if I… kill some bacteria that is bad I always also kill bacteria that's good. So I… um… so I try to really keep my body as natural as possible.

Some foods were described as “disgusting” and “dirty,” including processed foods, “diet” and “low fat” products, foods containing preservatives, chocolate, dairy, foods containing added sugar, and ready-made-meals. Homemade meals were described as “cleaner food.” Ironically, despite their extensive dietary restrictions, individuals suggested that too much control was unhealthy. “I think it's also unhealthy to be too controlling about what you eat” (Silvia).

This rigid dietary control extended into individuals' choices of work, holiday destinations and places of residence. For example, Matt described his choice of part-time work as a carer as enabling him to maintain strict meal times.

That job helps my… my eating patterns and stuff, because I have… I do like morning, afternoon, and evening shifts. I do it every single day. So, like, yesterday I had a full day. So I worked from 7 to 11 [a.m.], and then I had from 11[a.m.] to 1 [p.m.] off, so that was my period time to eat, so… so… I'll eat before… I'll eat after I get back, so I'll eat nothing in the morning, I wake up, get back at 10 or 11, I'll eat then, and then I'll go back at 1 [pm] and stay until half two, eat at half two and then I'll go back at 5 [p.m.] and I'll eat at five before I go and I'll be out until 10 [p.m.]. My job actually helps, it helps me eat more than… if I did a full time job where you work from 8 to about half five, they are not gonna be happy with me stopping every 3 h to eat, so this job is probably better for me.

Other participants chose holiday destinations based on the local foods of the country. Most participants were physically active and places of residence were chosen based on the vicinity of parks to maintain their exercise routines. Although the degree of importance placed on physical activity varied, all participants were very specific about the type, intensity, and duration of their exercise sessions. For most, physical activity helped to achieve or maintain a particular body shape. Matt was exercising to “get bigger,” Cat and Silvia took ballet classes to maintain a lean body shape, while Sarah engaged in long distance running for the same reason. Physical activity was instrumental in their motivation to control physical appearance. In addition, individuals implicated their body as an indicator of what healthy is. “If your body is happy, then, you know, I think that is like… healthy” (Lynn). Participants indicated that the body is an entity that needs to be listened to and be taken care of; “listen to your body I guess, and see how the body reacts” (Cat).

### Ethical Considerations

Ethical considerations for the environment, food production practices and animal welfare played a key role in maintaining dietary restrictions. Individuals defined themselves as supporters of ethical food producers. This theme is related to a change in participants' perceptions described earlier in “Journey.” In particular, food was perceived as something which must be consumed for survival, rather than enjoyment. Em suggested that “killing animals is like selling your soul,” further explaining that adding an ethical dimension to the reasons for following her diet made it easier to avoid transgressions.

When you change for ethical reasons it is a really strange question because like a… why do I miss killing animal? You know? I mean when I became for social reasons or health reasons, yeah! I did miss the taste of meat. But then when now, because I changed like… my perception has changed because of watching all this vegan content.

Four participants reported following a vegan diet, one individual was vegetarian and one participant adhered to a halal diet. Although it was acceptable for social acquaintances not to share the same values as participants regarding food, ethical concern for food production was a common characteristic between the varying diets. For example, Ebou followed the tradition of his Muslim faith:

Different people kill it [animal] in different ways, so if you sort of tase an animal and then kill it that way, the actual animal blood won't drain properly, so that's the whole point in sort of sacrificing it in a certain way because the blood all comes out from the shock and adrenaline, it sort of… it's a much cleaner way of killing that animal.

Rafaela expressed distrust about the food's journey from producers to the market shelf: “everything needs to be organic, I know where it comes from, I can source it and have the insurance that my food has not been tampered with.” Motivation for eating “healthy” was grounded not only in the quest for purity and particular body shape, but also in a desire to minimise harm to animals and support producers transparent with consumers about their practices.

## Discussion

This paper presents an analysis of experiences of individuals with possible ON, and their reasons for following their dietary patterns, and aimed to better understand the development of this elusive condition. Consensus on the development of recognised eating disorders is that the causes are complex and multifactorial (Polivy and Herman, 2002), but little is known about the development and classification of ON.

BRT aims to understand and predict behaviour and suggests that while the likelihood of an occurrence of a certain behaviour relies on intentions, global motives, beliefs and values held about this behaviour, reasons “for” and “against” represent a key dimension (Westaby, 2005). Interestingly, this study shows the change in beliefs about food from the values held by participants' families to new ones reflecting personal autonomy. Reasons our participants provided for adherence to their diet were distributed across four themes; Journey, Social, Rules/control, and Ethical considerations. The variety of justifications provided contribute to the difficulty in defining ON and were motivated by social influences, desire for control during life changes, concerns for ethical food production, animal welfare, weight maintenance, and had moral undertones. These show similarities to factors perceived to contribute to the development of ON by Dutch mental health professionals, with societal transitions and control being considered critical factors (Syurina et al., 2018). Interestingly in our study, there was disparity observed in participants' dietary intakes and psychometric scores (see Figures 1, 2). Taken together, these findings suggest ON presents idiosyncratically and that it is important for the reasons underlying the development of the behaviour to be examined.

The first theme (Journey) reflected the participants' perceptions of the factors that contributed to their current dietary status. Particularly interesting were the perceptions of what healthy eating meant to the individuals; it was identified as an assertion of “agency” over their lives. “Agency” can be described as how strongly a person believes s/he can impact on experiences and behaviours and has been suggested to influence success of psychotherapeutic treatment for established ED (Kristmannsdottir et al., 2019). Our participants described the development of their eating preferences as an attempt to establish independence from their family's food beliefs and a mechanism to assert control over their lives in times of change (e.g., moving to another country). Striving for control is not uncommon among individuals with ED. For example, a cognitive behavioural theory of AN suggests that the extreme need to control food consumption stems from perceptions of failure in other aspects of one's life (Fairburn et al., 1999). In this case, control over eating provides individuals with immediate evidence of successfully executed behaviour and overall self-control that may be more difficult to obtain in other areas (e.g., work, relationships) (Syurina et al., 2018). Indeed, ON has been suggested to represent a coping behaviour in patients with AN (Barthels et al., 2017). Perhaps the quest for control is similar in these eating pathologies but control over the quantity of consumed food shifts to its quality. It is unclear whether this shift in cognition is beneficial to treatment of AN, and more research is needed.

Four participants in this study had occupations (fashion models) that place great value on physical appearance. Despite the fact that the proposed diagnostic criteria for ON mention weight loss only as a secondary outcome (Dunn and Bratman, 2016), participants stated that maintaining a particular body shape was one of their reasons for adherence to a self-defined healthy diet. For these individuals the quest for control over eating may have been further reinforced by the fact that their professional success was directly dependent on their appearance. Healthy eating and physical activity were described as means to achieve and maintain the desired physique. Like dancers and athletes, fashion models are considered a risk group for disturbed eating behaviour (Treasure et al., 2008). After years of using clinically underweight models in the fashion industry, several countries adopted restrictions on employment of individuals with Body Mass Index below 18 kg/m^2^ in an attempt to tackle EDs (Sykes, 2017). While it is not known whether placing these restrictions has led to any changes in ED rates in this population, it may have impacted on the image individuals in the industry attempt to convey to the rest of the society (Syurina et al., 2018). Stereotypes attributed to individuals with ED are well-explored. With society's eye on the fashion industry as the breeding ground for eating pathologies, individuals involved might be motivated to present themselves favourably and avoid self-descriptions that would compromise their image; a healthy diet could represent a socially acceptable way to justify their dietary restrictions. Vartanian (2015) suggested that dietary adherence can be used to make a particular impression on others based on the stereotypes associated with those eating behaviours e.g., people consider eaters of “good food” more likeable, feminine, and attractive (Steim and Nemeroff, 1995). The assertion of our participants that they follow a “healthy diet” may be an attempt at impression-management based on their food consumption. From this perspective, eating behaviours reflective of possible ON can be adopted to transmit a particular image extending beyond what one eats and positioning one as a person that stands for ethical practices in food production and advocates for animal welfare, both reasons (ethical considerations) individuals in this study provided for their dietary behaviours.

All of our participants talked about social influences on the development of their dietary patterns. The similarity of the accounts lies in the fact that the inspiration for the change derived from participants' families and acquaintances and was further reinforced by social media influencers, literature on popular diets, and documentary films about animal welfare. Similar findings were observed in a recent study by McGovern et al. (2020). Individuals' movement toward ON tendencies was initiated by reading nutrition articles on the internet, and influenced by health professionals and family members. Proposed diagnostic criteria for ON (Dunn and Bratman, 2016) state that this condition has a negative impact on individuals' social lives potentially leading to isolation. Participants in this study did not report feeling isolated due to their dietary restrictions, instead preferring to socialise with people following similar diets. Social influence in the context of eating behaviour has been extensively studied (Robinson, 2015). For example, people tend to consume less food when dining with someone eating very little (Roth et al., 2001). The complexity of eating behaviour emerges from the fact that it is not only reliant on hunger and satiety, but represents a social ritual in many cultures and people often model food behaviours to match perceived social norms. Individuals' preference to socialise with people adhering to similar dietary restrictions can lead to creating new social norms among a segregated group of people, promoting a sense of belonging. Dunn and Bratman (2016) observed a trait not included in the diagnostic criteria which might be helpful in identifying ON cases; the moral judgement of others based on their diets. This was characteristic of some of the accounts offered in the current study. However, the same concept has been discussed in relation to vegans and vegetarians suggesting that adherents to these diets use it to justify moral superiority over others (Kroeze, 2012). Five individuals in this study reported adhering to vegan/vegetarian diets, therefore it is not surprising that the accounts reflect moral overtones related to social relations and ethical considerations.

Control patterns for males and females appeared to be different, with males' desire to control food intake being closely related to either their desire to maintain a certain physique or to support their workout sessions, while females' control over food intake was motivated by an increased identification with the image some dietary patterns transmit to others. Whilst some research suggests ON may be more prevalent in females (Syurina et al., 2018), ours suggests it may present differently in males. Putative gender differences in the stated reasons should be explored in more detail, as this study only had two male participants.

This study is the first to identify that a preoccupation with appearance, quest for control and social aspects of participants' lives are reported as key reasons for adherence to self-defined healthy diets indicative of ON. This is a critical first step to inform the development of diagnostic criteria and determine the classification of ON, and crucially to mitigate harm from extreme dietary restrictions arising from obsessive healthy eating practices. Understanding the reasons for such behaviours and the possible psychological drivers behind their adoption and maintenance is a further step to aid identification of individuals at risk for developing ON.

In this study, participants were selected on the basis that they exhibited behaviours and attitudes reflective of possible ON. This is necessary while research builds a better understanding of the condition, but it must be acknowledged that given the lack of diagnostic tools, no study, including the present one, can claim to have investigated a sample of individuals diagnosed with ON. Recruitment was therefore based on the available evidence, to reflect possible ON. Another limitation was that men were underrepresented in this study, and including a more balanced sample would provide an opportunity to explore gender differences of ON experiences. Future studies should focus their efforts on finalising a robust definition of the condition and developing a diagnostic tool, facilitating identification of participants for future research. Qualitative studies such as this one, which have explored the experience of individuals following a restricted diet, will facilitate in this development. In line with existing research (Syurina et al., 2018; Walker-Swanton et al., 2020), the present study identified social and cultural influences as an important domain in development of ON tendencies. Future studies should explore this in more detail. It would be particularly informative to explore whether ON, like established eating disorders, runs in families.

## Conclusion

Several reasons for the onset and maintenance of ON tendencies were identified in this study. External social influences were described as one of the reasons for the onset of ON tendencies, yet social isolation resulting from dietary choices was not reported. Participants preferred to socialise with those who shared their dietary preferences, suggesting ON had an organising rather isolating role in their lives. In addition, control and choices of a particular dietary intake manifested the need for establishing agency and identity particularly at times of change, suggesting life transitions may be a risk factor. This study also suggests that reasons for adhering to a “healthy” diet are far more complex than the desire for purity. Better understanding of the reasoning behind food choice will help to inform the education of nutrition specialists and mental health practitioners and in establishing whether this condition can be classified with Exias in the Diagnostic and Statistical Manual of Mental Disorders.

## Data Availability Statement

The raw data supporting the conclusions of this article will be made available by the authors, without undue reservation.

## Ethics Statement

The studies involving human participants were reviewed and approved by Kingston University Faculty of Science, Engineering and Computing Research Ethics Committee. The patients/participants provided informed consent verbally to participate in this study.

## Author Contributions

AP conceived and designed the study and oversaw the data collection, as well as the analysis and the writing of the paper. EM carried out the data collection and analysis and wrote the initial draft of the paper. EP supervised the data analysis and the writing of the paper. HM contributed to the writing of the paper. All authors critically reviewed the manuscript and approved the final version submitted for publication.

## Conflict of Interest

The authors declare that the research was conducted in the absence of any commercial or financial relationships that could be construed as a potential conflict of interest.
