# Supplementary material for: Using Behavioural Reasoning Theory to Explore Reasons for Dietary Restriction: A Qualitative Study of Orthorexic Behavioural Tendencies in the UK
Source: Front Psychol. 2021 Jul 8;12:685545. doi: 10.3389/fpsyg.2021.685545 (PMC8295483; doi:10.3389/fpsyg.2021.685545)
Supplement: Supplementary file 1 [file Table_1.DOCX]

Table 1.

*Consolidated criteria for reporting qualitative research (COREQ) (Tong et al., 2007).*

| No | Item | Guide questions/descriptions | Answers |
| --- | --- | --- | --- |
| Domain 1: Research team and reflexivity  Personal Characteristics | | | |
| 1 | Interviewer/facilitator | Which author/s conducted the interview or focus group? | First author. |
| 2 | Credentials | What were the researcher’s credentials? *E.g. PhD, MD* | The first author was completing a PhD Psychology degree and has an MSc in Health Psychology. |
| 3 | Occupation | What was their occupation at the time of the study? | Student. |
| 4 | Gender | Was the researcher male or female? | Female. |
| 5 | Experience and training | What experience or training did the researcher have? | The first author was supervised by the second author, who had previously published qualitative interview research. In addition, she attended training (“Summer School on Doing and Communicating Qualitative Research”) provided by Kingston University. |
| Relationship with participants | | | |
| 6 | Relationship established | Was a relationship established prior to study commencement? | Seven participants were acquainted with the first author from personal and professional relationships. One participant was recommended by another participant because of the similarity of their dietary patterns. Two other participants were known to the second and the fourth authors from professional contacts. |
| 7 | Participant knowledge of the interviewer | What did the participants know about the researcher? *E.g. personal goals, reasons for doing the research* | All participants knew that this study was carried out as part of the first author’s PhD. All participants knew that results of this study may be published in an academic journal. |
| 8 | Interviewer characteristics | What characteristics were reported about the interviewer/facilitator? *E.g. Bias, assumptions, reasons and interests in the research topic* | None |
| **Domain 2: study design**  Theoretical framework | | | |
| 9 | Methodological orientation and Theory | What methodological orientation was stated to underpin the study? *E.g. grounded theory, discourse analysis, ethnography, phenomenology, content analysis* | Thematic analysis. Reported on page 6 under the “Data analysis” section.  In addition, this study utilised Behavioural Reasoning Theory to explore participants reasons for development and adherence to the self-defined healthy diet. Reported on page 3. |
| Participant selection | | | |
| 10 | Sampling | How were participants selected? *E.g. purposive, convenience, consecutive, snowball* | The recruitment strategy involved purposeful snowball sampling technique. Participants were invited to participate in this study based on the observations of eating behaviours and attitudes made by the research team. |
| 11 | Method of approach | How were participants approached? *E.g. face-to-face, telephone, mail, email* | The first contact was made via telephone with eight and via email with two participants. |
| 12 | Sample size | How many participants were in the study? | Ten participants took part in the study. |
| 13 | Non-participation | How many people refused to participate or dropped out? Reasons? | Ten participants were contacted and all ten voluntarily took part in the study. |
| 14 | Setting of data collection | Where was the data collected? *E.g. home, clinic, workplace* | Eight interviews were carried out in a location of participants’ choosing. E.g. private room in a library, classroom at the University.  Two interviews were conducted via Skype because participants were away. |
| 15 | Presence of non-participants | Was anyone else present besides the participants and researchers? | Nobody else was present. |
| 16 | Description of sample | What are the important characteristics of the sample? *E.g. demographic data, date* | The study included eight females and two males. Participants’ demographic data are reported in Table 1. |
| Data collection | | | |
| 17 | Interview guide | Were questions, prompts, guides provided by the authors? Was it pilot tested? | The interviewer used the prepared interview schedule, which was approved by the Research Ethics Committee. Interviews were semi-structured. Participants were encouraged to explore questions for as long as they wanted. Interviewer only asked clarifying questions when answers were unclear or needed clarification.  The study was not pilot tested. |
| 18 | Repeat interviews | Were repeat interviews carried out? If yes, how many? | Repeat interviews were not carried out. |
| 19 | Audio/visual recording | Did the research use audio or visual recording to collect the data? | All interviews (Skype and face-to-face) were audio recorded using the “AudioNote” app on the iPhone 6 plus. |
| 20 | Field notes | Were field notes made during and/or after the interview or focus group? | Field notes were made during the interviews. |
| 21 | Duration | What was the duration of the interviews or focus group? | Duration of the interviews ranged from 40 to 120 minutes, with mean length of approximately 60 minutes. The total recording was 8.08 hours. |
| 22 | Data saturation | Was data saturation discussed? | Data saturation was discussed. |
| 23 | Transcripts returned | Were transcripts returned to participants for comment and/or correction? | Transcripts were not returned to participants. |
| **Domain 3: analysis and findings**  Data analysis | | | |
| 24 | Number of data coders | How many data coders coded the data? | The first author was the primary coder. Codes and themes were reviewed by the second author and revised. Final themes were reviewed by all authors. |
| 25 | Description of the coding tree | Did authors provide a description of the coding tree? | Authors did not provide a description of the coding tree. |
| 26 | Derivation of themes | Were themes identified in advance or derived from the data? | Themes were derived from the data. |
| 27 | Software | What software, if applicable, was used to manage the data? | Atlas.ti (ATLAS.ti Scientific Software Development GmbH) software was used for open thematic coding. |
| 28 | Participant checking | Did participants provide feedback on the findings? | Participants did not provide feedback on the findings. |
| Reporting | | | |
| 29 | Quotations presented | Were participant quotations presented to illustrate the themes/findings? Was each quotation identified? *E.g. participant number* | Pseudonyms were used to present quotations in the results section. |
| 30 | Data and findings consistent | Was there consistency between the data presented and the findings? | Data and findings were consistent with each other. |
| 31 | Clarity of major themes | Were major themes clearly presented in the findings? | Yes. |
| 32 | Clarity of minor themes | Is there a description of diverse cases or discussion of minor themes? | Yes. |
